# The Pathophysiology of HIV-/HAART-Related Metabolic Syndrome Leading to Cardiovascular Disorders: The Emerging Role of Adipokines

**DOI:** 10.1155/2012/103063

**Published:** 2011-12-08

**Authors:** John Palios, Nikolaos P. E. Kadoglou, Stylianos Lampropoulos

**Affiliations:** ^1^2nd Department of Cardiology, “Attikon” University Hospital of Athens, Haidari 12462, Greece; ^2^Department of Cardiology, “Bodosakio” General Hospital of Ptolemaida, Ptolemaida 50200, Greece

## Abstract

Individuals infected with human immunodeficiency virus (HIV) frequently demonstrate metabolic syndrome (MS) associated with increased incidence of cardiovascular disorders. Characteristics of HIV infection, such as immunodeficiency, viral load, and duration of the disease, in addition to the highly active antiretroviral therapy (HAART) have been suggested to induce MS in these patients. It is well documented that MS involves a number of traditional cardiovascular risk factors, like glucose, lipids, and arterial blood pressure abnormalities, leading to extensive atherogenic arterial wall changes. Nevertheless, the above traditional cardiovascular risk factors merely explain the exacerbated cardiovascular risk in MS. Nowadays, the adipose-tissue derivatives, known as adipokines, have been suggested to contribute to chronic inflammation and the MS-related cardiovascular disease. In view of a novel understanding on how adipokines affect the pathogenesis of HIV/HAART-related MS and cardiovascular complications, this paper focuses on the interaction of the metabolic pathways and the potential cardiovascular consequences. Based on the current literature, we suggest adipokines to have a role in the pathogenesis of the HIV/HAART-related MS. It is crucial to understand the pathophysiology of the HIV/HAART-related MS and apply therapeutic strategies in order to reduce cardiovascular risk in HIV patients.

## 1. Introduction

Treatment with highly active antiretroviral therapy (HAART) in patients infected with human immunodeficiency virus (HIV) has been documented to significantly increase life expectancy [1]. However, adverse metabolic effects like dyslipidemia, increased blood pressure, and insulin resistance have been attributed to HAART [2, 3]. Therefore, the use of HAART raises concerns regarding metabolic disorders and cardiovascular risk in HIV-infected patients who now present an extended life expectancy. An increase of approximately 26% of the risk for myocardial infarction has been reported in patients on HAART [4]. The detrimental effect of HAART on the arterial wall properties [5–8] has been proposed as an underlying mechanism, while it has been documented that HIV infection per se may promote atherosclerosis through immunodeficiency, chronic inflammation progress, viral load, and endothelial cell dysfunction, and either directly or indirectly via metabolic risk factors [9–13]. The exact pathophysiological events underlying the development of metabolic changes in HIV-infected patients are still under investigation. Several studies have identified specific defects in adipocyte function as main drivers in the pathogenesis of some of the metabolic changes in these patients. The list of adipocyte-secreted cytokines, known as adipokines, has been continuously expanded to include biomolecules, such as leptin, adiponectin, resistin, visfatin, apelin, acylation stimulating protein, omentin, and vaspin. In addition to this, TNF-*α*, adipose-derived interleukins, and acute-phase proteins have been also considered as adipokines by some researchers [14]. We should mention that the current terminology refers to a cytokine as an immunomodulating agent. Taking this into account, adiponectin, leptin, and resistin are not appropriately considered as adipokines, since they do not act on the immune system. Nevertheless, these peptides are still referred to as adipokines in the literature; however, they could be more accurately put into the larger, growing list of adipose-tissue-derived hormones. The role of those adipose-tissue-secreted hormones in the pathophysiology of HIV-/HAART-related metabolic syndrome (MS) in HIV-infected patients is still the subject of intense research. Therefore, we decided to review published data regarding the emerging role of adipokines in the increased cardiovascular risk in HIV-infected patients related to the HIV/HAART-associated MS. The main differences between the pathogenesis of MS in HIV-infected patients and other patients are summarized in Table 1.

## 2. Body Fat and Waist Circumference Abnormalities in HIV-Infected Patients

The prevalence of the HIV-associated “lipodystrophy” syndrome according to previous studies approaches 80% of patients receiving HAART [15], while other studies report only a prevalence of 17% [16]. Significant differences in “lipodystrophy” rates exist when comparing patients with or without HAART. In one of these studies the prevalence of any body change was 62% in protease inhibitor (PI)-experienced patients, 33% in PI-naive patients and 21% in antiretroviral-naive patients [17]. There seems to be a lower prevalence of morphological body shape and fat redistribution changes in HIV-infected children, while an increase in serum adipokine levels has been additionally described [18]. According to the European Paediatric Lipodystrophy Group, approximately a quarter of children and adolescents show signs of lipodystrophy, similar to those described in adults [19]. 

Lipoatrophy typically includes decreased subcutaneous fat in the upper or lower extremities with prominent veins, loss of buttock subcutaneous fat, and facial atrophy [20]. The fat wasting should be differentiated from other wasting conditions associated with HIV infection, including the AIDS-wasting syndrome, malnutrition, cachexia, adrenal insufficiency, and severe chronic infections. 

Lipodystrophy is characterized by lipoatrophy/fat loss, lipohypertrophy/fat accumulation, or both [20]. Fat accumulation can be seen around the neck, the dorsocervical region as “buffalo hump,” the abdomen, and the trunk or as subcutaneous fat deposits, that is, lipomas, particularly in the dorsocervical area. These findings can be either symmetric or asymmetric. Breast enlargement has also been observed.

## 3. Dyslipidemia and Insulin Resistance in HIV-Infected Patients

The association of dyslipidemia with many antiretroviral regimens and especially PIs has been well established [21]. The effect on total cholesterol levels appears to be regimen dependent as shown in the Swiss HIV-1 Cohort Study [22]. Potential mechanisms for PI-associated dyslipidemia include (a) inhibition of sterol regulatory element-binding protein-1 (SREBP-1) activation in the liver and/or adipocytes along with the protease-mediated breakdown of apolipoprotein-B [23], (b) direct enhancement of the formation of very-low-density lipoproteins (VLDLs) [24] and the reduction of lipoprotein lipase activity [25], and (c) changes in the mobilization of lipid stores [26]. Nevertheless, lipid disorders can also occur during therapies not including PIs [27, 28]. Insulin resistance is also a significant metabolic side effect associated with HAART. PIs affect insulin sensitivity through various mechanisms such as IRS-1 phosphorylation and subsequent glucose uptake from adipocytes [29]. Lipodystrophy may also result in B-cell dysfunction [30] and is associated with impaired feedback of insulin on B-cells [31]. On the other hand, HIV-1 infection itself may be independently linked to the attenuation of insulin sensitivity. The HIV-1 accessory protein Vpr induces transcription of glucocorticoid-responsive promoters, *in vitro*, thus increasing sensitivity to glucocorticoids [32]. It also attenuates peroxisome-proliferator-activated receptor-*γ* (PPAR-*γ*) activity [33] and interferes with the suppressive effects of insulin on forehead transcription factors [34]. Therefore, it contributes to the tissue-selective insulin resistance. The end result of all the described factors is the attenuation of insulin sensitivity.

## 4. Parameters of HIV-/HAART-Induced Metabolic Syndrome and Cardiovascular Disorders

Concerning arterial stiffness, expressed by pulse wave velocity (PWV), and markers of metabolic profile, we recently compared HIV-infected patients age- and sex-matched individuals with either with hypertension or without any chronic disease [35]. In that study, HIV-infected patients had higher PWV levels than healthy controls, but lower than hypertensive patients. Notably, patients on HAART had similar PWV to hypertensive patients. In multivariate analysis, the independent determinants of increased arterial stiffness were HAART duration and MS parameters, like serum lipids and blood pressure. 

In our previously published study, we performed a comparative evaluation of endothelial dysfunction between HIV-positive individuals and age- and sex-matched controls with similar risk factors and a group of patients with established coronary artery disease (CAD). HIV-infected patients presented endothelial dysfunction to a similar extent as patients with CAD. Moreover, HIV-infected patients taking PIs had higher blood pressure, cholesterol, and triglycerides than those not taking PIs. Importantly, endothelial dysfunction was associated with elevated serum triglycerides. Therefore, we concluded that HAART-induced hypertriglyceridemia might have been a plausible mechanism explaining endothelial dysfunction in HIV-infected individuals. In the same study, we found an increased carotid intima media thickness (IMT), an index of subclinical carotid atherosclerosis, in HIV-infected patients. Most importantly, carotid IMT levels were equivalent in HIV-infected and CAD groups. So, we suggested that subclinical carotid atherosclerosis was closely related to PI-related changes of metabolic parameters in HIV-infected patients. 

Current recommendations by the National Cholesterol Education Program for HIV-infected persons focus on LDL-C levels, as the primary target of the lipid-lowering therapy. The LDL cholesterol goal has been set <160 mg/dL for persons with 0-1 cardiovascular risk factors, <130 mg/dL for persons with multiple (2+) risk factors, and <100 mg/dL for persons with established coronary heart disease (CHD) or CHD risk equivalents. After lifestyle modifications, statins should be used to lower LDL-C levels. Therapy with fibrates is recommended to lower triglycerides levels. However, omega-3 fatty acids can be effective means of triglycerides lowering as well, particularly in patients with markedly elevated triglycerides levels. The efficacy of statins in HIV-infected persons appears to be lower than expected, although adherence to statins therapy has not been well assessed. Statins combining high potency and minor interactions with antiretroviral therapy (pravastatin, fluvastatin, atorvastatin, and rosuvastatin) should be preferred as the initial therapy, though comparative studies in HIV-infected persons are scarce. 

Adequate choice and dosing of lipid-lowering drugs, given as single agents or in combination therapy, and care for drug compliance in HIV-infected patients at moderate or high cardiovascular risk should help maximize their long-term health.

## 5. HIV-/HAART-Induced Metabolic Syndrome: The Role of Adipokines

Visceral adipose tissue (VAT) is the predominant adipose tissue compartment responsible for the production of adipokines. A growing body of evidence supports the emerging role of adipokines in metabolic homeostasis and atherosclerosis. In this paper, we have reviewed the recent progress regarding the role of adipokines in the HIV/HAART-induced MS and cardiovascular disease (CVD). A better understanding of the molecular mechanisms will lead to the discovery of new drugs and reduce the incidence of lipodystrophy and related metabolic complications in HIV-infected patients receiving HAART. 

### 5.1. Leptin

Leptin, which was the first adipokine identified, influences food intake through direct effects on the hypothalamus [36]. This adipocyte-derived hormone has actions in the brain (e.g., hypothalamus, cortex, and limbic areas) and in a number of peripheral tissues as well as cells of the pancreas, liver, and immune system. The central actions of leptin include energy and glucose homeostasis, reproductive functions, and immunity [37, 38]. The relationship between adiposity and leptin levels appears similar to controls and HIV infected but untreated patients [39]. On the other hand, severe lipodystrophy syndromes are characterized by loss of subcutaneous adipose tissue and a relative deficiency of leptin [40]. The effect of HAART on leptin levels is subject of controversy. In few studies, HAART administration had been associated with lipodystrophy and hypoleptinemia [41], while numerous studies had predominantly demonstrated no effect of HAART on leptin concentrations [42]. The above discrepancy was mainly attributed to the differential effects of HAART on fat-mass distribution and not directly to leptin per se [43]. Indeed, HAART without fat-mass re-distribution did not influence leptin levels [44]. Moreover, there is a weak correlation of leptin with insulin sensitivity in HIV-infected population [45].

Accumulating data support the proinflammatory and proatherogenic properties of leptin in either noninfected or HIV-infected patients [46, 47]. Although there is no prospective study evaluating the association of leptin with long-term cardiovascular events in HIV infected patients, the high levels of leptin in that population apparently increases the inflammatory milieu and perhaps the total cardiovascular risk. Future studies will elucidate the role of leptin in CVD progression in HIV-infected patients.

### 5.2. Adiponectin

Adiponectin, a well-studied adipokine, is secreted by fatty cells and is widely regarded to exert a counterregulatory role in atherogenesis, by its antioxidant, anti-inflammatory, antithrombotic, and direct anti-atherosclerotic properties [48]. Adiponectin expression is suppressed in patients with obesity and type 2 diabetes, showing an inverse relationship with insulin resistance and visceral adiposity [49]. Treatment-naïve, HIV-1-positive patients appear with suppressed adiponectin levels [50]. In previous studies, circulating adiponectin levels were suppressed in patients with chronic HIV infection and fat redistribution, but the underlying mechanisms remain obscure [51, 52]. Moreover, patients treated with HAART, especially those with lipodystrophy, showed gradual downregulation in adiponectin serum levels [53]. Notably, in the latter subgroup of patients, the HAART-induced hypoadiponectinemia was associated with accelerated cardiovascular impairment [54]. Taken all together, the suppression of adiponectin levels in HIV-infected patients under HAART may deteriorate numerous metabolic parameters (e.g., insulin resistance, lipid profile, etc.) leading to detrimental cardiovascular events.

### 5.3. Resistin

Despite the quite promising data from rodent studies, human data did not consistently confirm the association of resistin with insulin resistance, diabetes, and obesity [55]. Contrary to the aforementioned findings, elevated resistin levels have been found in HIV-infected patients compared to uninfected individuals. That difference was ascribed to HAART-related metabolic changes [56, 57]. Perhaps, HIV/HAART-related MS alters the regulatory mechanisms of resistin, but this hypothesis requires further investigation. On the other hand, the predominant sources of human resistin are macrophages and mononuclear leukocytes, and to a lesser extent, adipocytes [58]. Conditions of low-grade systemic inflammation, such as diabetes and atherosclerosis, may induce macrophage expression of resistin and increase circulating levels, independently of metabolic changes. The latter notion is also supported by the previously reported contributory role of resistin to pathologic processes, like inflammation, endothelial dysfunction, thrombosis, and smooth muscle cell dysfunction, leading to CVD [59]. Unambiguously, future studies will shed more light on the interplay between resistin and HIV/HAART-related MS and CVD.

### 5.4. Visfatin

Visfatin, also known as nicotinamide phosphoribosyltransferase (NAMPT), functions as a growth factor for early B cells within the immune system [60]. Although visfatin is expressed and regulated by the adipose tissue, its relationship with adiposity-related insulin resistance is controversial [61, 62]. Regarding the impact of HAART on visfatin, a single study demonstrated significantly increased serum visfatin levels after HAART initiation, along with insulin resistance augmentation, and without concomitant changes in fat mass [63]. Thus, the fluctuations of insulin resistance and glucose homeostasis in HIV-positive patients may explain the regulation of visfatin. 

Importantly, patients with stable coronary and carotid disease appear with high circulating levels of visfatin [64, 65], while macrophages derived from human unstable carotid and coronary plaques increasingly express visfatin [66]. Future studies clarifying the involvement of proinflammatory visfatin in the HIV/HAART-associated MS may better define the cardiovascular risk.

### 5.5. Apelin

Apelin, an adipocyte-secreted factor, has been recently identified as a contributor to glucose homeostasis and insulin resistance [67]. Abundant expression of apelin and its receptor, APJ, has been detected in endothelial cells from large arteries and coronary blood vessels and in the heart [68]. Moreover, previous trials have suggested apelin as a potent regulator of cardiovascular function [69]. We and other investigators have recently documented the inverse relationship between circulating apelin levels and CHD [70, 71]. 

The interplay between apelin with HIV infection and initially reported by Zou et al. who described the inhibition of HIV-1 and HIV-2 entrance in CHO and NP-2 cells expressing CD4 and its receptor after preincubation with apelin [72]. Moreover, apelin receptor has been shown *in vitro* to act as HIV-1 coreceptor [73]. Taken together, more functional studies are required to determine the precise role of apelin/APJ in cardiovascular regulation, insulin resistance, and the susceptibility to HIV infection. This information would help to evaluate its potential as a future drug target.

### 5.6. Vaspin

A novel adipokine, vaspin, has been recently designated as a mediator of obesity, insulin resistance, and type 2 diabetes [74]. Both animal and clinical studies suggest that elevated vaspin levels in serum and adipose tissue may be a compensatory response to elevated insulin resistance, secondary to metabolic complications [75]. Extremely limited data implicate the association of low serum vaspin levels with atherosclerosis development and progression [64, 76]. Although vaspin exerts insulin-sensitizing and atheroprotective actions, its relationship with cardiovascular complications in HIV/HAART-related MS has not been investigated.

## 6. Conclusions

Adipokines appear to have a leading role in the pathogenesis of the HIV/HAART-related MS [77]. Leptin deficiency and hypoadiponectinemia, for example, correlate with insulin resistance and body fat abnormalities. These disorders affect the cardiovascular health of HIV patients through the amelioration of atherosclerosis and endothelial dysfunction. Furthermore, novel adipokines, such as visfatin, apelin, and vaspin, have emerged as potential mediators of the interplay between MS and atherosclerosis in HIV-infected patients. It is of great interest to study the pathological mechanism of the HIV/HAART-related MS and its cardiovascular complications and try to apply therapeutic strategies in order to reduce cardiovascular risk in HIV patients.

## Figures and Tables

**Table 1:** The main differences between the pathogenesis of MS in HIV-infected patients and other patients.

	HIV-infected patients	Non-HIV-infected patients
(A)	HAART-induced dyslipidemia, hypertriglyceridemia, HDL reduction, especially if PI used	Fat abnormal metabolism leading to hypertriglyceridemia and dyslipidemia

(B)	HAART-induced leptin deficiency and hypoadiponectinemia leading to insulin resistance	Hypoadiponectinemia leading to insulin resistance and abnormal glucose metabolism

(C)	HIV-associated “lipodystrophy” syndrome—body fat abnormalities—fat accumulation around the neck, dorsocervical region as “buffalo hump,” abdomen, and trunk	Waist circumference enlargement due to abdominal fat accumulation

## Abbreviations

AIDS: Acquired immunodeficiency syndrome
CHD: Coronary heart disease
CHO: Chinese hamster Ovary
CVD: Cardiovascular disease
HIV: Human immunodeficiency virus
HAART: Highly active antiretroviral therapy
IMT: Intima media thickness
LDL: Low-density lipoprotein
MS: Metabolic syndrome
NAMPT: Nicotinamide phosphoribosyltransferase
NNRTI: Non-nucleoside reverse transcriptase inhibitors
NP: Neural progenitors
NRTI: Nucleoside reverse transcriptase inhibitors
PI: Protease inhibitors
PPAR-*γ*: Peroxisome-proliferator-activated receptor-*γ*
PWV: Pulse wave velocity
SREBP: Sterol regulatory element-binding protein
TNF: Tumor necrosis factor
VAT: Visceral adipose tissue
VLDL: Very-low-density lipoprotein.
